# A Uremic Pig Model for Peritoneal Dialysis

**DOI:** 10.3390/toxins14090635

**Published:** 2022-09-14

**Authors:** Joost C. de Vries, Maaike K. van Gelder, Anneke S. Monninkhof, Sabbir Ahmed, Diënty H. M. Hazenbrink, Tri Q. Nguyen, Gèrard A. P. de Kort, Evert-Jan P. A. Vonken, Koen R. D. Vaessen, Jaap A. Joles, Marianne C. Verhaar, Karin G. F. Gerritsen

**Affiliations:** 1 Department of Nephrology and Hypertension, University Medical Centre Utrecht, 3508 GA Utrecht, The Netherlands; 2 Department of Pharmaceutical Sciences, Utrecht University, 3584 CG Utrecht, The Netherlands; 3 Department of Pathology, University Medical Centre Utrecht, 3508 GA Utrecht, The Netherlands; 4 Department of Radiology, University Medical Centre Utrecht, 3508 GA Utrecht, The Netherlands; 5 Central Laboratory Animal Research Facility, Utrecht University, 3584 CJ Utrecht, The Netherlands

**Keywords:** animal model, kidney failure, peritoneal dialysis, standard peritoneal permeability assessment, mass transfer area coefficient

## Abstract

With increasing interest in home dialysis, there is a need for a translational uremic large animal model to evaluate technical innovations in peritoneal dialysis (PD). To this end, we developed a porcine model with kidney failure. Stable chronic kidney injury was induced by bilateral subtotal renal artery embolization. Before applying PD, temporary aggravation of uremia was induced by administration of gentamicin (10 mg/kg i.v. twice daily for 7 days), to obtain uremic solute levels within the range of those of dialysis patients. Peritoneal transport was assessed using a standard peritoneal permeability assessment (SPA). After embolization, urea and creatinine concentrations transiently increased from 1.6 ± 0.3 to 7.5 ± 1.2 mM and from 103 ± 14 to 338 ± 67 µM, respectively, followed by stabilization within 1–2 weeks to 2.5 ± 1.1 mM and 174 ± 28 µM, respectively. Gentamicin induced temporary acute-on-chronic kidney injury with peak urea and creatinine concentrations of 16.7 ± 5.3 mM and 932 ± 470 µM respectively. PD was successfully applied, although frequently complicated by peritonitis. SPA showed a low transport status (D/P creatinine at 4 h of 0.41 (0.36–0.53)) with a mass transfer area coefficient of 9.6 ± 3.1, 4.6 ± 2.6, 3.4 ± 2.3 mL/min for urea, creatinine, and phosphate respectively. In conclusion, this porcine model with on-demand aggravation of uremia is suitable for PD albeit with peritoneal transport characterized by a low transport status.

## 1. Introduction

A uremic large animal model for preclinical testing of novel peritoneal dialysis (PD) therapies is urgently needed. An ideal uremic large animal model would bear a close resemblance to humans and be characterized by stable uremia with uremic toxin concentrations comparable to patients with ESKD, without requiring (long-term) maintenance dialysis to minimize animal discomfort and costs. Previously described uremic large animal models include goat, sheep and porcine models. However, none of these models meet the above-mentioned criteria, as they either have severe uremia (e.g., bilateral nephrectomy) resulting in dialysis dependency and limited survival [1,2,3] or only mild uremia, which is less comparable to the situation in dialysis patients [4,5]. In addition, few studies have performed PD in a large animal model, and peritoneal membrane transport characteristics were not routinely assessed [6,7,8,9].

The aim of this study was to establish a translational porcine model with (temporary) uremic toxin concentrations in the range of patients with ESKD for in vivo evaluation of novel PD therapies. Our first objective was to induce chronic kidney disease (CKD) by bilateral subtotal renal artery embolization. Subsequently, we aimed to temporary aggravate uremia by post-embolization administration of gentamicin to obtain uremic toxin levels comparable to those in ESKD. Our final objective was to determine peritoneal transport characteristics by means of the standard peritoneal permeability assessment (SPA). To our knowledge, this is the first study that reports detailed peritoneal transport characteristics in a uremic pig model.

## 2. Results

### 2.1. Renal Artery Embolization

**Figure 1:**
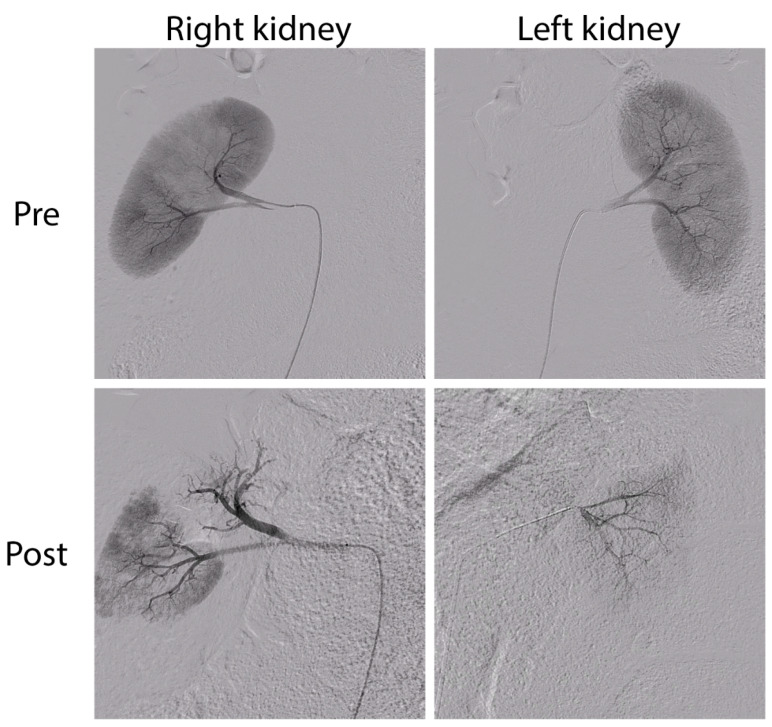
Contrast arteriogram before (Pre, **top**) and after (Post, **bottom**) embolization of the right (left panels; subtotal embolization) and left (right panels; complete embolization) kidney.

Bilateral subtotal renal artery embolization was performed in seven pigs. In the first pig, ~20% of total kidney tissue was spared, determined visually during the procedure by the interventional radiologist using contrast angiography. A control renal arteriogram was performed 43 days post-embolization (due to insufficient degree of CKD), which demonstrated unimpeded homogenous perfusion of the partially embolized kidney, and incomplete perfusion of the completely embolized kidney. Re-embolization sparing ~15% of total kidney tissue was found to be more effective at creating chronic kidney injury and was thus applied in the following embolization procedures (Figure 1).

Embolization induced acute kidney injury (Table 1), with peak urea and creatinine concentrations of 7.5 ± 1.2 mmol/L (*p* = 0.002 vs. baseline) and 338 ± 67 µmol/L (*p* = 0.004 vs. baseline), respectively, 2 to 3 days post-embolization. Partial recovery occurred with stabilization of urea and creatinine plasma concentrations at 2.5 ± 1.1 mmol/L (*p* = 0.17 vs. baseline) and 174 ± 28 µmol/L (*p* = 0.03 vs. baseline), respectively. Bicarbonate, phosphate, potassium and calcium were not influenced by embolization (Table 1). Glomerular Filtration Rate (GFR), measured with iohexol [10], was 61.5 (61.0–66.3) mL/min/1.73 m^2^ (median (IQR)) before embolization, which decreased to 46.7 ± 11.7 mL/min/1.73 m^2^ after 78 ± 33 days following embolization.

### 2.2. Survival and Complications

Of the seven pigs that underwent embolization, one pig was euthanized 1 day post-operatively and another died 2 days post-operatively because of hemoptysis with respiratory insufficiency. These animals had been stable during the procedure and directly afterwards. A rapid clinical deterioration with severe dyspnea and discomfort was observed 1 to 2 days after embolization. Post-mortem investigation showed diffuse pulmonary hemorrhage, neutrophil infiltration and minor pulmonary embolism in both animals. PVA embolization particles were not detected in the lungs and a clear cause could not be identified. Some indications of complement activation were observed (Figure S1). Since we considered a PVA-related reaction as a realistic possibility, we changed the type of embolization beads to Embosphere^®^ Microspheres. No adverse reactions occurred in the three animals embolized thereafter. Median follow-up of the pigs that survived the embolization was 7.6 months (range: 4.4–9.4), after which the animals were euthanized because the body weight was >100 kg which is too high to be representative for the human situation (i.e., a scientific endpoint). Besides elective peritoneal dialysis experiments that were performed during the window with gentamicin-induced acute-on-chronic kidney injury, no life-sustaining dialysis treatment was needed and plasma creatinine concentrations remained stable or slightly increased in time (Supplemental Figure S2). The animals were in good clinical condition and—except during gentamicin-induced acute-on-chronic kidney injury—body weight gradually increased (Supplemental Figure S3).

### 2.3. Gentamicin-Induced Acute-on-Chronic Kidney Injury

**Figure 2:**
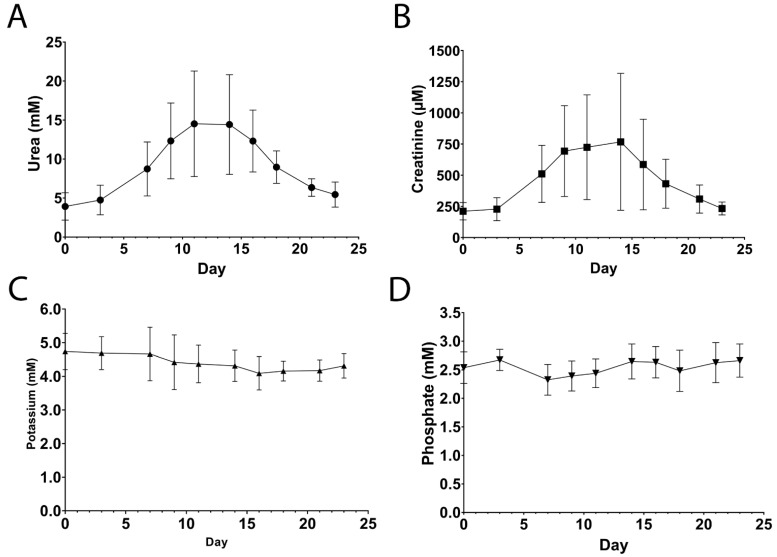
Urea (**A**), creatinine (**B**), potassium (**C**) and phosphate (**D**) plasma concentrations after administration of gentamicin (day 0). Data are presented as mean ± SD, *n* = 10 administrations in *n* = 5 animals. Linear imputation was used for missing plasma concentrations.

**Figure 3:**
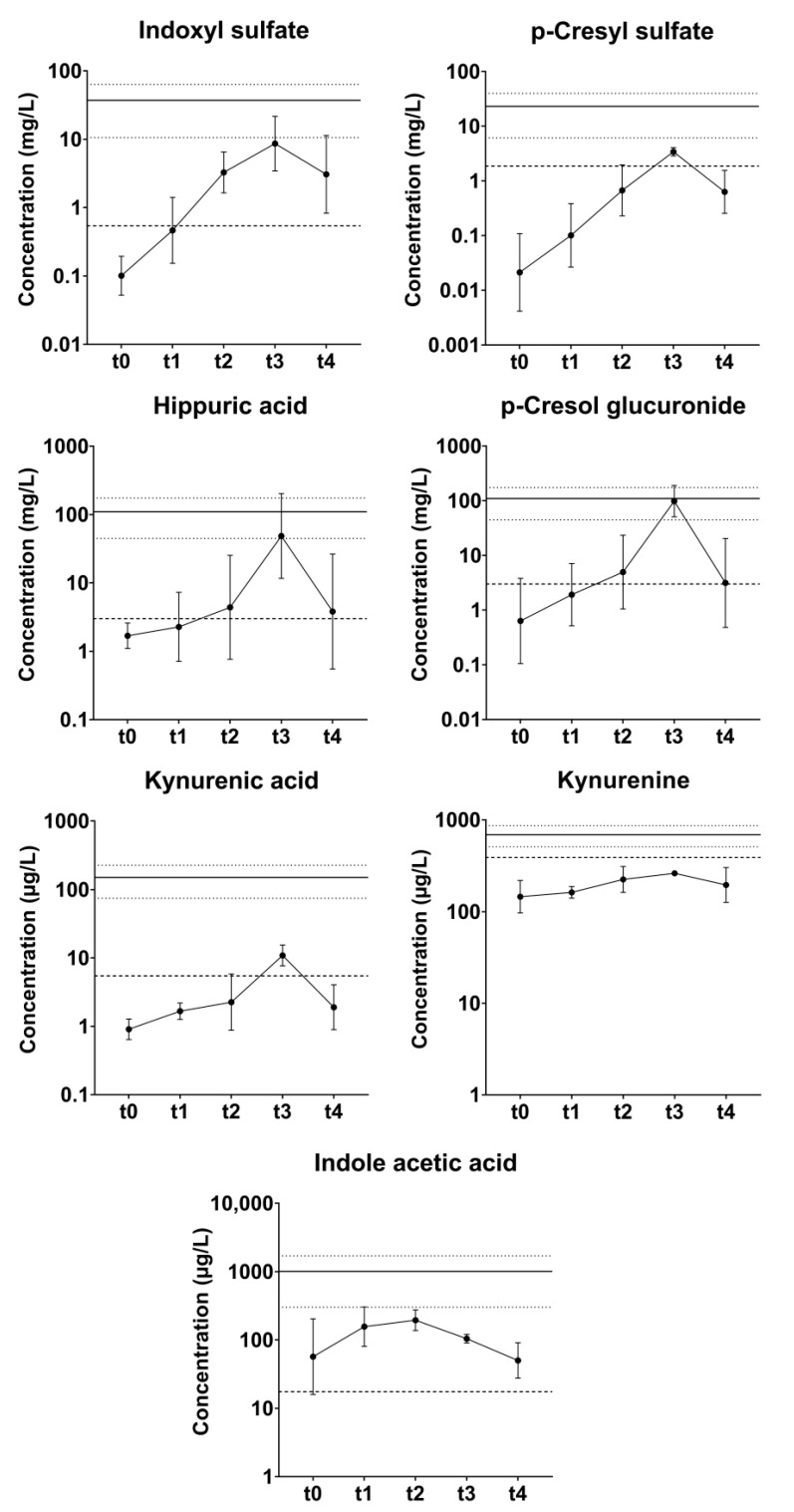
Plasma concentrations of protein bound uremic toxins (PBUTs) pre-embolization (*n* = 5, t0) and during gentamycin-induced acute kidney injury in pigs (*n* = 5, pre-gentamicin (t1); *n* = 4, week 1 (t2); *n* = 2, week 2 (t3); *n* = 5, week 3+ (t4)). Data are presented as mean ± SD. The striped line denotes the average concentration in healthy human adults, whereas the solid line and dotted lines denote the mean and SD, respectively, in ESKD patients [11,12].

Acute-on-chronic kidney injury was induced by administration of gentamicin 10 times (2 episodes per pig). During four episodes (all in different animals), the pigs showed clinical and biochemical signs of dehydration requiring intravenous fluids for 1–3 days (1–4 L total volume administered). Together with temporary decreased appetite, this resulted in reversible weight loss. One animal experienced transient disorientation/ataxia for 2–3 days, possibly due to the ototoxic effect of gentamicin. Plasma urea and creatinine concentrations increased from 3.6 ± 0.9 mM to 16.7 ± 5.3 mM (*p* = 0.02) and from 212 ± 58 µM to 932 ± 470 µM (*p* = 0.04), respectively, whereas potassium and phosphate remained stable (Table 1, Figure 2 and Figure S4). Peak values were achieved at 12 ± 1.8 days after start of the administration of gentamicin (i.e., ~5 days after discontinuation) for urea and at 11 ± 1.7 days for creatinine. After reaching peak values, a slow return to (near-) baseline values was observed, which generally took > 28 days (since start of gentamicin). In addition to creatinine and urea, protein bound uremic toxin (PBUT) plasma concentrations were temporarily elevated during gentamicin-induced acute kidney (Figure 3).

### 2.4. Urinalysis

Urine portions were collected before and after embolization, as well as during the first episode of acute-on-chronic kidney injury (Table 2). Proteinuria was already present at baseline. Although absolute protein concentrations were variable and did not significantly change, the protein to creatinine ratio seemed to increase with increasing kidney failure. 

### 2.5. Reduction of Plasma Bicarbonate Concentration

**Figure 4:**
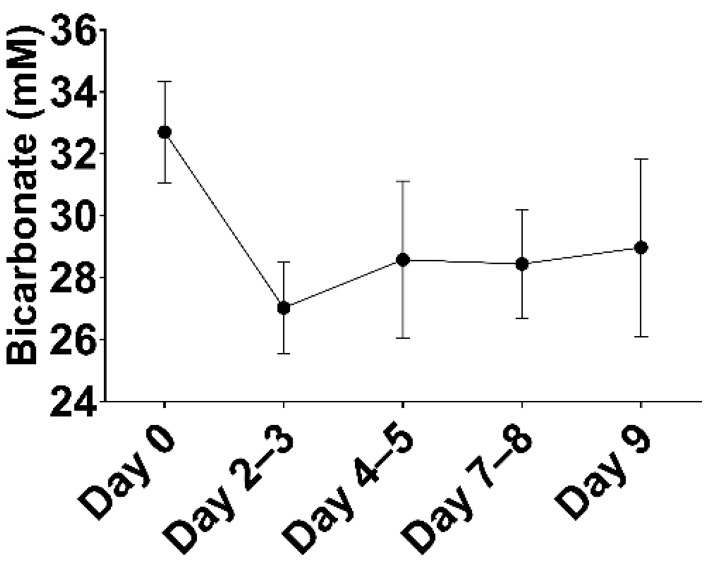
Effect of ramipril and acetazolamide on plasma bicarbonate concentrations. Data are presented as mean ± SD (*n* = 8).

Plasma bicarbonate concentrations in the pigs were relatively high (32.9 ± 1.3 mM) as compared to humans (22–29 mM). Subtotal renal artery embolization and gentamicin administration did not significantly influence bicarbonate concentrations (Table 1). To achieve bicarbonate concentrations more comparable to those observed in PD patients [13], a combination of acetazolamide and ramipril was administered, which resulted in a decrease in plasma bicarbonate concentrations after ~3 days from 32.7 ± 1.6 mM to 27.0 ± 1.5 mM (*p* < 0.0001; Figure 4), after which bicarbonate concentrations gradually tended to increase again (29.0 ± 2.9 mM), although this varied considerably per animal. 

### 2.6. Peritoneal Transport

A total of 20 SPA experiments were performed in five pigs. Peritoneal transport parameters are depicted in Table 3. MTACs, clearance, and UFV could not be calculated in three SPAs due to inaccurate residual volume measurements. MTACs and clearance rates were relatively low as compared to those reported in humans (Table 3) [14,15,16]. In accordance with this, D/P ratios for creatinine after 4 h were low (0.41 (0.36–0.53); median (IQR)) and D/D0 ratios for glucose after 4 h were high (0.65 (0.58–0.70)), indicating a low peritoneal transport status in the pigs. Net ultrafiltration was 160 ± 322 mL in 4 h. 

Despite broad antibiotic treatment and hygienic precautions, all pigs suffered from peritonitis during one or more SPA experiments (*n* = 7). During peritonitis, a significant increase in MTAC was observed for most solutes (urea, creatinine, phosphate) except potassium, but this was not accompanied by a significantly higher clearance (Table 3), which is probably due to net absorption of fluid (−117 ± 300 mL) coinciding with a lower D/D0 for glucose at 4 h (0.57 ± 0.15). D/P ratios for urea and creatinine were higher, corresponding to a higher transport status (low-average during peritonitis versus low in absence of peritonitis).

### 2.7. Histopathology

**Figure 5:**
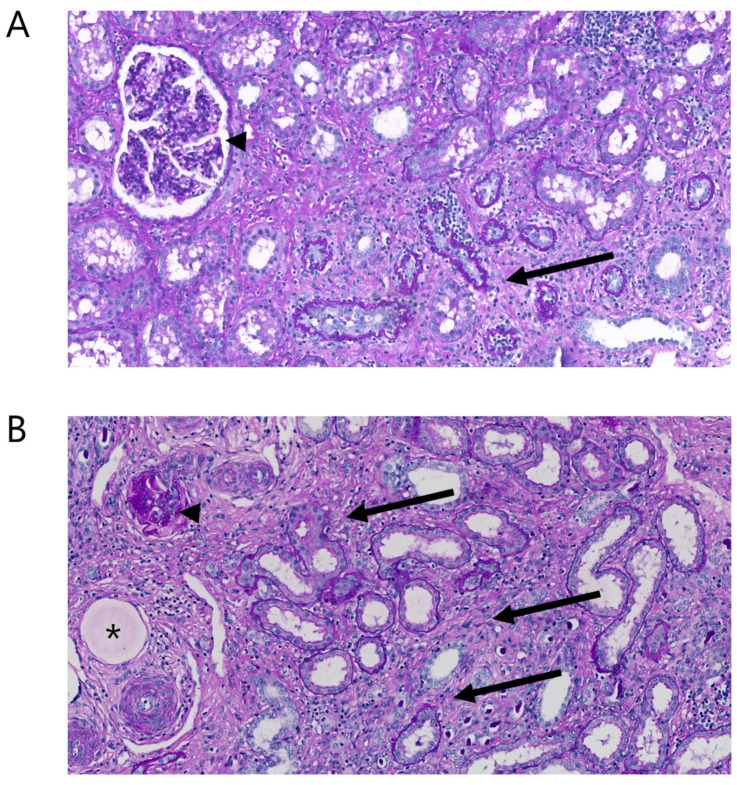
Morphology of kidney tissue ~4 months after subtotal embolization. (**A**) In a non-embolized area of the kidney, mild to moderate tubular atrophy and fibrosis is seen (arrows), as well as a normal glomerulus (arrow head). (**B**) In an embolized area, severe tubular atrophy and fibrosis is seen (arrows), as well as a globally sclerosed glomerulus (arrow head). The asterisk depicts an embolization bead.

In all animals, kidney biopsies showed diffuse chronic injury in the non-embolized parts of the kidneys (Figure 5A). Interstitial fibrosis, tubular atrophy, and glomerulosclerosis were observed in many areas and the overall degree of interstitial fibrosis and tubular atrophy was estimated at ~30%. Embolization particles were seen throughout the embolized areas (Figure 5B).

**Figure 6:**
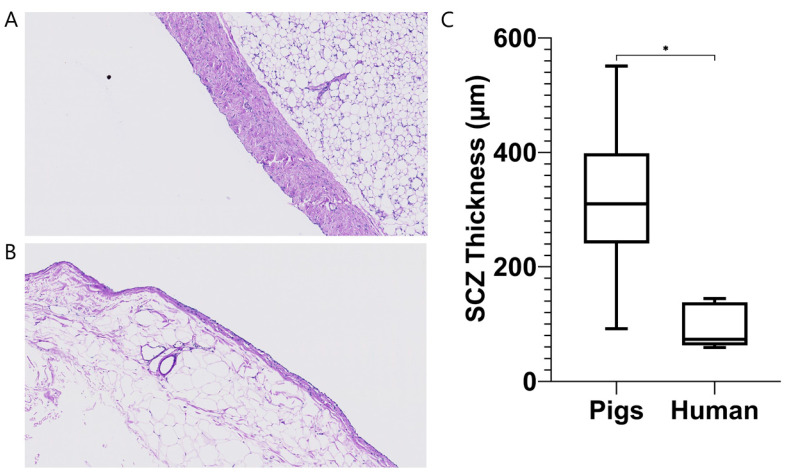
Peritoneal biopsies of pigs (**A**) and (uremic) humans (**B**) reveal a thicker submesotheial compact zone (SCZ) in pigs (**C**). Data are presented as mean ± SD and range, *n* = 6 (pigs) and *n* = 5 (humans). The data from the human subjects were published elsewhere [17,18]. * *p* < 0.05.

Analysis of peritoneal biopsies revealed a significantly thicker submesothelial compact zone in uremic pigs (316.9 ± 146.7 µm, *n* = 6) compared to humans (95.1 ± 40.0 µm, *n* = 5; *p* = 0.01) (Figure 6).

## 3. Discussion

We present a novel porcine uremic model that can be aggravated on-demand and used for peritoneal dialysis. Bilateral subtotal nephrectomy (through embolization) induced stable moderate CKD. Administration of gentamicin subsequently effectively induced temporary acute-on-chronic kidney injury with a uremic solute profile (urea, creatinine, phosphate, indoxyl sulfate and hippuric acid) that resembled that of dialysis patients [11,12]. PD could be performed with peritoneal transport characteristics indicative of a low transport status.

A previous study in pigs showed mild chronic kidney injury (increase in creatinine from ~65 to ~105–175 µM) with removal of 60 to 75% of total kidney mass, moderate chronic kidney injury (increase in creatinine from ~65 to 175–425 µM) with removal of 75 to 90% of total kidney mass, and lethal kidney injury (if untreated) when >90% of kidney mass was removed (increase in creatinine from ~65 to ~970 µM with death after 10 and 16 days) [4]. In line with this, Misra et al. showed moderate chronic kidney injury in pigs when ~75% of total kidney mass was removed (increase in creatinine from ~112 to ~210 µM and urea from ~3 to ~7 mM) [3]. In another porcine model moderate chronic kidney injury was induced through bilateral renal artery stenosis (copper coils) and high-cholesterol diet. This resulted in a ~70% stenosis, 41% decrease in renal blood flow, 33% loss of kidney mass, and a subsequent increase in creatinine from 112 ± 6 to 203 ± 6 µM and urea from 1.7 ± 0.1 to 2.4 ± 0.2 mM [5]. Since we aimed for a stable uremic model with high uremic solute plasma concentrations but no need for maintenance hemodialysis, we initially chose to remove (embolize) ~80% of kidney tissue, but this proved to be insufficient to induce chronic high uremic solute plasma concentrations (increase in creatinine from 93 µM to 143 µM and urea remaining stable at 1.4 mM). Embolization with preservation of ~15% of total kidney tissue subsequently resulted in stable moderate chronic kidney injury without the need for maintenance dialysis. Of note, the response varied per animal, so regular monitoring of biochemical parameters, in particular directly after embolization, is recommended. Throughout the follow-up period, animals were clinically stable and showed stable creatinine concentrations (except for the episodes with gentamicin-induced acute-on-chronic kidney injury), which allows for CKD research with a long-term follow-up.

Embolization was generally well-tolerated, with the notable exception of two pigs in whom respiratory insufficiency occurred 1–2 days after subtotal renal artery embolization. Histological examination of the lungs showed pulmonary hemorrhage, neutrophil infiltration and pulmonary embolism but no PVA particles in the lungs, which makes it unlikely that (particle-related) pulmonary embolism was the cause of death. Direct complement activation causing leukocyte activation and subsequent pulmonary sequestration of leukocyte aggregates may have played a role. Although this phenomenon, called complement activation-related pseudoallergy (CARPA), has not been described for in vivo application of PVA particles, polymers carrying surface hydroxyl groups (such as PVA) have been shown to activate the complement system in vitro through the alternative pathway [19,20]. In addition, there are reports of severe complement activation-related anaphylactoid reactions (CARPA) in pigs after intravenous infusion of polymeric nanoparticles which can lead to cardiopulmonary distress and, in severe cases, be lethal [21]. Pigs appear to be particularly sensitive for this reaction. After switching to alternative embolization material (tris-acryl gelatin microspheres (Embosphere^®^)), which was well-tolerated in mini-pigs in another study [22], we did not observe these complications anymore.

While post-embolization creatinine concentrations were comparable to humans with CKD (212 ± 67 µM), urea concentrations remained remarkably low (3.6 ± 1.4 mM) despite a high protein diet (~2.7–5.3 g/kg/day), which has been shown to increase urea concentrations in pigs [23]. Of note, urea reference values in pigs (1.5–7.5 mM) [24,25] are slightly lower than in humans (3–8 mM), but even taking this into account, urea levels during CKD were surprisingly low. Relatively low urinary urea concentrations (~100 mM, see Table 3) suggest low urea production—assuming that urea is primarily (≥80%) excreted via the urine and urine production is <2.5 L/day—despite high-protein diet. Possibly, urea production is low due to a state of net protein synthesis (anabolic state) which is in line with their fast increase in body weight. Misra et al. and Chade et al. also reported low urea concentrations (2.4 ± 0.2 mM and ~7 mM, respectively) in the stable phase after induction of kidney injury. Significant uremia with urea concentrations within the range of those of dialysis patients could be induced with gentamicin, making dialysis research possible during these episodes of acute-on-chronic kidney injury.

Gentamicin successfully induced reversible acute-on-chronic kidney injury with plasma solute concentrations comparable to ESKD. As gentamicin induces tubular cell damage and necrosis [26,27], this might result in tubular loss of phosphate and potassium, resulting in hypophosphatemia and hypokalemia. This appeared not to be the case and both phosphate and potassium were stable during gentamicin-induced acute-on-chronic kidney injury, while there was a significant increase in creatinine, urea, and PBUT concentrations. Of note, phosphate concentrations, while within the normal range for pigs, were well within the human uremic range (>2 mM, both in healthy and uremic situation). There was a significant increase in creatinine, urea, and PBUT concentrations. The increase in creatinine and urea, i.e., the degree of acute-on-chronic kidney injury, was quite variable however, and close monitoring of both biochemical parameters as well as animal welfare is essential after gentamicin administration to ensure animal welfare.

PD in pigs was performed with ease and transport parameters could be measured by means of a SPA. On average, pigs have a (very) low transport status compared to humans, the D/P ratio for creatinine at 4 h being 0.41 (0.36–0.53) in pigs versus a mean of 0.77 in humans [28]. In accordance with this, MTAC for creatinine was low, namely, 3.8 (2.8–5.5) mL/min versus 9.4–10.16 mL/min [15,28]. These differences may, at least in part, be due to the thickness of the porcine peritoneum compared to human peritoneum, which increases diffusion distance and thus diffusion resistance. Of note, lack of a reliable intraperitoneal volume marker (dextran 70 is no longer commercially available) precluded measurement of effective lymphatic absorption rate (ELAR), which may have led to slight underestimation of MTAC. Unfortunately, all pigs developed peritonitis at some (or multiple) time(s) during follow-up, which resulted in a (transient) higher transport status (low average). Peritonitis was most likely due to the inherent unhygienic nature of housing in a pig pen (albeit according to EU regulations). Tunneling of the catheter to the back, above the spine and prophylactic antibiotics, as we performed, could not prevent this. Of note, the pigs did not appear to experience noticeable discomfort during peritonitis. UF volume in the pigs averaged 160 mL for a 4 h 1.36% dwell, which is similar to values observed in humans (~85–300 mL) [28,29,30], even though the average D/D0 ratio was relatively high (0.65 (0.58–0.70); 0.13–0.56 in humans) [28].

Previous studies on PD in uremic pigs are difficult to interpret as most of them only report plasma levels of uremic toxins (no efficacy parameters) [31,32,33], or the degree of CKD is not reported [7,8]. Only Geary et al. reported efficacy parameters. Urea clearance was 2.2 ± 0.3 mL/min during two static 2 h dwells of 30 mL/kg in pigs of ~12 kg. When normalized for body weight this clearance appears higher than what we observed in our study (urea clearance 5.8 ± 2.1 mL/min during a 4 h dwell of 2 L in pigs of 40–100 kg). To our knowledge, our study is one of the most comprehensive reports on a uremic pig model for PD to date, as the degree of renal failure, the peritoneal transport characteristics and the efficacy of the ensuing PD are well-described. In addition, our model offers various advantages. First, subtotal renal artery embolization provides a relatively simple way of inducing renal failure with relatively little discomfort and a short recovery time for the animal. Second, the resulting degree of CKD does not require chronic dialysis and does not result in uremic symptoms in the animal. This allows for long-term follow-up, repeated experiments in a single animal, improved animal welfare (as opposed to chronic severe uremia), and reduced costs. Third, reversible on-demand acute-on-chronic kidney injury by gentamicin administration resulted in uremic solute concentrations in the range of (peritoneal) dialysis patients, thereby allowing for dialysis experiments representative for the human situation.

Our study is limited by a lack of data on blood pressure, protocolized and regular urinalysis, and measurements of middle molecules. Blood pressure measured on one of the legs with a cuff for humans was unreliable, and invasive blood pressure measurements were not available at the animal facility. Urine collection at fixed timepoints during the follow-up proved difficult, as even after multiple hours in a metabolic cage the animals sometimes did not urinate. As such, the costs—in terms of discomfort for the animal by standing solitary in the metabolic cage—did not outweigh the benefits of regular urine collection and was discontinued at an early stage. Measurement of several middle molecules (cystatin C, beta 2-microglobulin, vitamin B12) on routine laboratory diagnostic analyzers and with commercially available ELISA’s (for beta 2-microglobulin) did not provide usable results. Another limitation of our model is that not all biochemical parameters during stable CKD were representative of the human situation (e.g., relatively high bicarbonate, low PBUT concentrations). Furthermore, recurrent peritonitis—that could not be prevented by various measures (see above)—may limit the use of this model for monitoring long-term effects of PD on the peritoneal membrane.

In conclusion, we established a uremic pig model providing stable moderate CKD with the possibility for on-demand temporary aggravation of kidney failure to a degree comparable with end stage kidney disease in humans. The model allows for multiple dialysis sessions in the same animal over a prolonged period of time, therewith limiting the required number of animals. PD is well feasible, although peritoneal transport status of the pigs is low compared to humans and peritonitis could not be prevented. 

## 4. Materials and Methods

### 4.1. Experimental Animals

The study was approved by the Central Commission for Animal Experiments (CCD; AVD115002015226, Centale Commissie Dierproeven, The Hague, The Netherlands). and performed in accordance with national guidelines for the care and handling of animals. Healthy female Yorkshire pigs (Sus scrofa domesticus; Topigs Norsvin, Lelystad, The Netherlands) (*n* = 7) four months of age and weighing approximately 40 kg (at the time of embolization) were used. The animals were housed in walking pens with solid concrete floor covered with straw. Temperature of the animal room was maintained at 20–25 °C and artificial lighting was provided with a 12 h–12 h light/dark cycle. Animals were offered a low-calorie (~47–96 kcal/kg/d; depending on life stage), high-protein (~2.7–5.3 g/kg/day, depending on life stage) diet (Ssniff Spezialdiäten GmbH, Soest, Germany; V4133-000) to slow weight gain—and thus keep body weight within human range for a longer period of time—and promote uremia, respectively. Drinking water was provided ad libitum. Following PD and/or venous catheter placement, animals were individually housed to prevent dislodgement of these catheters by other animals. Both catheters were flushed daily with 0.9% saline and filled with heparinized glycerol to maintain catheter patency. Topical prophylactic antibiotics (Neopen; procaine pencillin and neomycin) were applied daily to prevent skin port infection of the catheters.

### 4.2. Embolization Procedure

Animals received premedication (ketamine 13 mg/kg, midazolam 0.7 mg/kg, atropine 0.05 mg/kg) and anesthesia was induced with propofol (3.5 mg/kg). Animals were subsequently intubated and maintained on propofol 3.5 mg/kg/h. Analgesia was administered preoperatively (remifentanil 0.03 mg/kg/h) and postoperatively (single dose meloxicam 0.4 mg/kg, 6 days BuTrans band aid 20 µg/h). A single dose of antibiotics (amoxicillin/clavulanic acid, 10 mg/kg) was administered to prevent infection. A 7 French sheath was placed in the femoral artery using Seldinger technique (Avanti + 6F, Cordis). Subsequently, the macrocatheter (Cobra glidecath 5F, Terumo) was inserted and advanced into the renal artery (for contrast fluid) and the microcatheter (Progreat 2.4F, Terumo) was used for the embolization. The kidney to be partially embolized was selected by the radiologist based on the observed anatomy in each animal. For the first four embolization procedures polyvinyl alcohol (PVA) particles (ContourTM PVA Embolization Particles, 45–150 microns, Boston Scientific) were used and for the latter three Embosphere^®^ Microspheres (Merit Medical, Maastricht, The Netherlands; comprised of tris-acryl polymer impregnated with porcine gelatin). The remaining area of perfused kidney tissue was visualized by contrast angiography. Directly after embolization, a venous catheter was placed in the internal jugular vein. Blood samples were drawn pre- and post-embolization until uremia stabilized.

### 4.3. Peritoneal Catheter Placement

A PD-catheter (Straight Tenckhoff 47 cm, single cuff. Argyle, Covidien™, Dublin, Ireland) was surgically inserted into the peritoneal cavity of the animals by an experienced animal technician. Animals received premedication (ketamine 13 mg/kg, midazolam 0.7 mg/kg, atropine 0.05 mg/kg) and were placed under general anesthesia with propofol (3.5 mg/kg). Animals were subsequently intubated and maintained on propofol 3.5 mg/kg/h. Analgesia was administered preoperatively (remifentanil 0.03 mg/kg/h) and postoperatively (single dose meloxicam 0.4 mg/kg). A BuTrans band aid 20 µg/h was placed one day prior to the PD catheter placement and continued for 6 days. An incision of ~5 cm was made in the flank of the animal in the midaxillary line, approximately 10 cm rostral from the rear legs. From the incision, a subcutaneous tunnel was made towards the spine and a small incision was made at the end of the tunnel. After spraying antibiotics (Depomedrol) in the subcutaneous tunnel, the catheter was pulled through. Then, a small incision was made in the abdominal wall and the catheter was carefully inserted towards the pelvis. Catheter function was assessed before closing the abdominal wall with a purse string suture, after which catheter function was assessed again. If the catheter functioned well, the two skin incisions were closed, a MiniCap Extended Life PD Transfer Set (Baxter, Deerfield, IL, USA) was put on the catheter, which was then filled with a heparin/glycerol lock solution. Initially, all animals were treated with amoxicillin/clavulanic acid 10/1 mg/kg twice daily and daily neopen drops to the skin port to prevent peritonitis. However, the amoxiclav was discontinued due to limited effect on preventing peritonitis.

### 4.4. Administration of Gentamicin for Aggravation of Uremia

Acute-on-chronic kidney injury was induced prior to SPA measurements with twice-daily administration of gentamicin (10 mg/kg i.v.) for 7 days. If no increase of creatinine or urea was observed after 7 days, treatment with twice-daily gentamicin (10 mg/kg) was continued up to a maximum of 10 days. Blood samples were taken regularly before, during, and the weeks following gentamicin treatment to monitor uremia. Animals were observed daily for clinical signs of discomfort or adverse effects of the treatment. Based on the biochemical and clinical parameters (intake, weight), intravenous and/or intraperitoneal fluids (NaCl 0.9%) were administered in case of suspected dehydration. Emergency hemodialysis was available if severe uremia, severe hypervolemia or severe metabolic derangement would occur. Of note, this would only be applied in case of a realistic chance of spontaneous recovery thereafter, as ESKD requiring maintenance dialysis was considered a humane endpoint.

### 4.5. Kidney Function Measurements

Glomerular Filtration Rate (GFR) was determined before and after embolization (but not during gentamicin-induced uremia) as described by Van Gelder et al. [10,34]. In short, the pigs received a 5 mL bolus of iohexol (Omnipaque 300 mg/mL, GE Healthcare, Chigago, IL, USA) via the venous catheter in the jugular vein. Blood samples were obtained before the iohexol bolus, at the start, and after 10, 60, 120, 180, 210, 240, 300, 360, 480 and 1440 min, to measure iohexol concentrations and hematocrit. Plasma iohexol clearance, which represents GFR, was estimated by a three-compartment nonlinear mixed-effects model. GFR values were subsequently normalized to body surface area calculated with the Swindle/Kelley method [35].

### 4.6. Reduction of Plasma Bicarbonate Concentration

Plasma bicarbonate concentrations in the pigs were relatively high (±30–35 mM) as compared to human reference values (22–29 mM). In order to achieve bicarbonate concentrations more comparable with those observed in PD patients (~25 mM) [13], a combination of acetazolamide (1 qd 500 mg p.o.) and ramipril (1 qd 1.25 mg p.o.) was administered to decrease tubular reabsorption of bicarbonate and promote tubular reabsorption of hydrogen ions, respectively, and thus decrease plasma bicarbonate. Administration of ra-mipril and acetazolamide was performed during stable CKD (*n* = 5) and acute-on-chronic kidney injury (*n* = 3).

### 4.7. Standard Peritoneal Permeability Analysis (SPA)

Peritoneal transport was assessed with a SPA as described by Pannekeet et al. [28] SPAs were performed in the operation room and preceded by an overnight dwell with Physioneal (PN) 35 1.36% glucose or Extraneal (both Baxter, Utrecht, The Netherlands). After complete drainage of the overnight dwell, a dialysate sample was taken from the effluent dialysate for the measurement of urea, creatinine, phosphate, sodium, glucose, albumin, total protein, leukocytes and for microbial culture. A fresh 2 L bag of warm (±37 °C) Physioneal 35 1.36% was instilled. A sample of PD effluent was taken directly after instilment and at 10, 30, 60, 120, 180 and 240 min for measurement of urea, creatinine, phosphate, sodium, potassium, glucose, albumin and total protein. Samples were obtained by draining 100–200 mL of dialysate in a sterile drain-bag (Baxter, XMC4284) of which a small sample (2–7 mL) was obtained in an aseptic manner through the sampling port of the drain-bag. The remaining fluid was re-instilled. Concentrations of albumin at t = 0 (directly after instilment) and of the overnight dwell were used to calculate residual volume before the start of the SPA (Equation (1)). After 240 min, a complete drain was performed, and the peritoneal cavity was flushed with fresh dialysate. Concentrations of albumin in the drained dialysate and dialysate used for flushing were used to calculate residual volume after SPA. Venous blood samples were obtained at t = 0, 120, and 240 min for measurement of urea, creatinine, phosphate, sodium, potassium, albumin and total protein.

RV = (V_in_ × C_t0_)/(C_drain_ − C_t__0_) (1)

RV = residual volume, V_in_ = instilled volume of fresh dialysate, C_t0_ = intraperitoneal concentration of albumin at t = 0 after instilment of the fresh dialysate, C_drain_ = concentration of albumin in the drained dialysate.

Net effective ultrafiltration volume (UFV) was calculated using Equation (2).

UFV_net_ = V_t240_ + RV_t240_ + V_sample_ − V_t0_ (2)

UFV_net_ = effective ultrafiltration volume, V_t240_ = the volume of the complete drain at the end of the SPA, RV_t240_ = the residual volume in the peritoneal cavity after the complete drain at the end of the SPA, V_sample_ = the volume of dialysate removed throughout the experiment through sampling, Vt0 is the total intraperitoneal volume at the start of the SPA (ergo the sum of the residual volume of the overnight dwell and the instilled volume).

D/P ratios at 4 h were calculated for urea and creatinine and D/D0 ratios at 4 h for glucose. Among the parameters that describe peritoneal transport, these ratios are commonly used and respectively reflect the ratio of the solute concentration in the dialysate at 4 h versus that in plasma and the ratio of the glucose concentration at 4 h versus that at 0 h. Mass transfer area coefficient (MTAC)—defined as the theoretical maximal clearance that can be achieved with diffusive transport if the solute has not yet accumulated in the dialysate—of urea, creatinine, phosphate and potassium was calculated as described by Pannekeet et al. [28] using the formula according to Garred (Equation (3)) [36]. Total solute removal and clearance rates were calculated using Equations (4) and (5), respectively.

MTAC = (V_t_/t) × ln((V_0_ × (P − D_0_))/(V_t_ × (P − D_t_))) (3)

This is the modified Garred et al. formula, where MTAC = mass transfer area coefficient, V_0_ = instilled dialysate volume, V_t_ = volume of the drained dialysate at t = 240, D_0_ = dialysate concentration of the solute at t = 0, D_t_ = dialysate concentration of the solute at t = 240, and P = mean of plasma solute concentrations at t = 0, 120 and 240.

TSR = (C_t_ × V_t_) − (C_0_ × V_0_) (4)

where TSR = total solute removal, C_t_ = dialysate concentration at t = 240, V_t_ = intraperitoneal dialysate volume at t = 240, C_0_ = dialysate concentration at t = 0, and V_0_ = intraperitoneal dialysate volume at t = 0.

Cl = TSR/(C_av_/t) (5)

where Cl = clearance, TSR = total solute removal, C_av_ = mean plasma concentration, and t = elapsed time in minutes.

Peritonitis was defined as the presence of two of the following criteria: a positive microbial culture of peritoneal effluent, cloudy effluent and/or elevated leukocyte count (>0.1 × 10^9^/L) in the effluent (minimum 2 h dwell).

### 4.8. Biochemical Monitoring Pre- and Post-Embolization and during Peritoneal Dialysis

Analysis of biochemical and hematological parameters was performed in the laboratory of clinical chemistry and hematology of the University Medical Center Utrecht. Biochemical parameters were measured using a AU5800 routine chemistry analyzer (Beckman Coulter, Brea, CA, USA) up to 28 September 2019, after which an Atellica Solution Immunoassay & Clinical Chemistry Analyzer was used (Siemens Healthineers, Erlangen, Germany). For hematological parameters, the Cell-Dyn Sapphire was used up to March 2020, after which an Alinity HQ Hematology Analyzer was used (both Abbot, Chicago, IL, USA).

### 4.9. Liquid Chromatography-Mass Spectrometry (LC-MS)

PBUTs were measured during *n* = 5 episodes of gentamicin-induced kidney injury. Kynurenine, kynurenic acid (KA), indoxyl sulfate (IS), indole-3-acetic acid (IAA), hippuric acid (HA), acetic acid (Hac; LC-MS grade) were purchased from Sigma-Aldrich (St. Louis, MO, USA). P-Cresyl sulfate and p-Cresyl glucuronide were purchased from AlsaChim (Illkirch-Graffenstaden, France). Water (LC-MS grade), acetonitrile (CAN; HPLC-S grade) and methanol (HPLC grade) were obtained from Biosolve (Valkenswaard, The Netherlands). Formic acid (analytical grade) was obtained from Merck (Darmstadt, Germany). Ultrapure water was produced by a Milli-Q^®^ Advantage A10 Water Purification Systems (Merck, The Netherlands).

An Accela LC system (quaternary pump and autosampler), coupled to a TSQ Quantum Ultra triple quadrupole mass spectrometer with heated electrospray ionization (ESI) was used for this study. Equipment and software for controlling, data recording and processing (Xcalibur version 2.07) were supplied by Thermo Fischer Scientific (San Jose, CA, USA).

A Waters ACQUITY UPLC HSS T3 column (100 mm × 2.1 mm, 1.8 µm particles) combined with an ACQUITY UPLC HSS T3 VanGuard pre-column (5 mm × 2.1 mm, 1.8 µm particles) was used and kept at a temperature of 40 °C. Mixed isocratic and gradient elution at a flow rate of 0.45 mL/min was applied for analyte separation. In the mobile phase, solvent A consisted of 0.2% (*v/v*) Hac, and solvent B was acetonitrile. The final method was an initial isocratic composition of 5% B held for 1 min, followed by a linear increase to 15% B for the next 1 min, and to 20% B in another 1 min. Then, B was increased linearly to 80% over the next min to flush the column. To re-equilibrate, B was reduced back to the initial isocratic composition of 5% and was held for 2 min.

Plasma samples were processed prior to LC-MS analysis. A volume of 20 µL plasma or surrogate matrix was pipetted into a 1.5-mL Eppendorf tube. Ultra-pure water was used as a surrogate matrix for calibration curve and quality control samples. For the calibration curve and quality control samples, the 20-µL water sample contained the standards. Subsequently, 30 µL of cold (4 °C) ACN with internal standards d4-kynurenine, d5-kynurenic acid, 13C6-indoxyl sulfate, d5- indole-3-acetic acid, d7-p-cresyl sulfate, d7-p-cresyl glucuronide and d5-hippuric acid were added. Samples were vortexed for 5 min on a plate vortexer and centrifuged for 5 min at 4000 RPM. A 35-µL sample of the supernatant was then collected in 1-mL round-bottom well of a polypropylene 96-deep well plate and diluted with 200 µL of ultra-pure water. Finally, the plate was gently shaken before placing in the autosampler for analysis.

### 4.10. Middle Molecule Measurements

Vitamin B12, beta-2-microglobulin and cystatin C were analyzed using routine clinical chemistry analyzers (AU5800, Beckman Coulter, Brea, CA, USA up to 28 September 2019; Atellica Solution Immunoassay & Clinical Chemistry Analyzer, Siemens Healthineers, Erlangen, Germany; from 28 September 2019 onwards). While vitamin B12 could be measured in plasma, levels in dialysate were generally below the lower limit of detection and could thus not be used to calculate solute removal or clearances. Beta-2-microglobulin and cystatin C could not be detected with the routine clinical chemistry analyzers in porcine plasma. Several commercially available ELISA kits for beta-2-microglobulin (MBS2880294 and MBS1604456 My BioSource, San Diego, CA, USA; CBS-EL002486PI, Cusabio) and Cystatin C (CSB-E13282p, Cusabio, Houston, TX, USA) were performed according to the manufacturer’s instructions without obtaining reliable results.

### 4.11. Histology

When the humane or scientific endpoint had been reached, pigs were euthanized by a veterinarian by intravenous administration of an overdose of pentobarbital sodium (Euthanimal Alfasan, Woerden, The Netherlands). After termination, cross-sectional biopsies of the cortex and medulla were taken from both the infarcted and vital part of the kidneys, as well as biopsies of the parietal peritoneum. In the case of the two pigs who died after embolization, additional biopsies (lung, spleen) were also collected. Of each biopsy, one half was snap frozen in liquid nitrogen and stored at −80 °C, with the other half being fixated in 10% formalin. The formalin-fixed biopsies were processed by the pathology department of the University Medical Center Utrecht. Slides were stained with Hematoxylin-Eosin (HE) and/or Periodic acid-Schiff (PAS). Subsequently, an assessment of tubular/interstitial damage, presence of PVA particles and (degree of) fibrosis was made for the kidney biopsies by an experienced attending pathologist. The stained peritoneal biopsies were scanned/digitized using the NanoZoomer XR slide scanner and software (Hamamatsu Photonics, Hamamatsu, Japan), and additional images were acquired with a BX51 inverted microsope (Olympus, Tokio, Japan) with Olympus CellSense v1.18 software. Submesothelial Compact Zone (SCZ) thickness was measured at ≥5 random locations using Aperio ImageScope v12.4.3.5008 (Leica Biosystems, Wetzlar, Germany). Slides were independently analyzed by two observers, one of whom an experienced renal pathologist. Data from human peritoneal thickness for comparison were kindly provided by Dr. A.C. Abrahams (UMCU). These data were obtained by measuring SCZ in peritoneal biopsies (*n* = 5) from a previous study in patients who received a pre-emptive renal transplant [17,18]. All subjects provided written informed consent upon inclusion in the original study. C4d was stained using a commercially available antibody (Clone SP91, Rabbit monoclonal; Cell Marque, Rocklin, CA, USA) following local protocol.

### 4.12. Statistical Analysis

Descriptive statistics are reported as mean ± SD or, where indicated, median (interquartile range). Normality of data was assessed visually using Q-Q plots, as well as using the Shapiro–Wilk test. For differences between two groups, an unpaired *t*-test or a Mann–Whitney U test was applied for continuous data, depending on normality of the data. For repeated measures, a one-way repeated measures ANOVA (with Geisser-Greenhouse correction and Tukey post hoc correction) or a Friedman test (with Dunn’s correction) was used, depending on normality of the data. In case of a missing value for a specific time point, linear interpolation was performed. For the embolization and acute-on-chronic kidney injury data, the experimental unit was the animal, whereas for the peritoneal dialysis efficacy a single SPA was the experimental unit. Analyses were performed using GraphPad Prism v8.3.0 (GraphPad Software, San Diego, CA, USA). In all statistical tests, a two-tailed *p*-value < 0.05 was considered significant.

## Figures and Tables

**Table 1:** Blood measurements before and after embolization, and before and after gentamicin administration.

	Urea (mM)	Creatinine (µM)	Bicarbonate (mM)	Phosphate (mM)	Potassium (mM)	Calcium (mM)
Pre-embolization	1.6 ± 0.3	103 ± 14	32.9 ± 1.5	2.65 ± 0.35	4.0 ± 0.3	2.44 ± 0.11
Peak value post-embolisation	7.5 ± 1.2	338 ± 67	34.2 ± 0.7	2.67 ± 0.57	4.2 ± 0.2	2.47 ± 0.14
Pre-gentamicin	3.6 ± 0.9	212 ± 58	30.8 ± 2.4	2.54 ± 0.16	4.7 ± 0.6	2.55 ± 0.07
(Peak) value post-gentamicin	16.7 ± 5.3	932 ± 470	29.4 ± 4.0	2.60 ± 0.21	4.4 ± 0.6	2.61 ± 0.24
Recovery from gentamicin-induced AKI	4.4 ± 0.68	250 ± 53	31.3 ± 2.4	2.56 ± 0.13	4.5 ± 0.3	2.62 ± 0.06

Data are presented as mean ± SD. Of note, *n* = 5 for the pre- and post-embolization values, and *n* = 5 (5 × average of 2 replicates per animal) for pre-gentamicin, peak value and recovery.

**Table 2:** Urinalysis during different stages/degrees of renal insufficiency.

	Urea (mM)	Creatinine (mM)	Total Protein (g/L)	TP/Creatinine (mg/mmol)
Pre-embolization	96 ± 35	22.2 ± 3.2	0.91 ± 0.71	43.6 ± 36.9
Post-embolization	109 ± 27	14.5 ± 1.7	1.15 ± 0.83	74.1 ± 49.7
Gentamicin peak *	90 ± 28	8.5 ± 3.4	0.68 ± 0.17	114.6 ± 85.9

Data are presented as mean ± SD. *n* = 5 (pre-embolization), *n* = 4 (post-embolization), and *n* = 3 (gentamicin peak) due to difficulties with urine collection. * Urine sample taken at the peak of gentamicin-induced acute-on-chronic kidney injury (day ~15–16). TP: Total Protein.

**Table 3:** Peritoneal transport characteristics in absence and presence of peritonitis.

		Total	No Peritonitis	Peritonitis	*p*-Value
MTAC (mL/min) (mL/min/1.72 m^2^)	Urea	9.1 (7.3–10.9) (17)	8.3 ± 1.8 (12)	12.6 ± 3.7 (5)	0.06
		15.2 (12.8–19.3) (17)	14.0 ± 4.8 (12)	20.8 ± 7.8 (5)	0.05
	Creatinine	3.8 (2.8–5.5) (17)	3.3 ± 0.9 (12)	7.7 ± 2.9 (5)	0.03
		5.4 (4.6–8.4) (17)	5.4 ± 2.0 (12)	13.1 ± 7.0 (5)	0.002
	Phosphate	3.4 ± 2.3 (17)	2.3 ± 1.1 (12)	6.0 ± 2.2 (5)	<0.001
		5.6 ± 4.3 (17)	3.7 ± 2.3 (12)	10.1 ± 5.0 (5)	0.002
	Potassium	18.3 ± 4.1 (17)	18.6 ± 4.7 (12)	17.4 ± 2.3 (5)	0.60
		30.6 ± 10.9 (17)	31.0 ± 11.8 (12)	29.6 ± 9.6 (5)	0.081
Clearance (mL/min) (mL/min/1.73 m^2^)	Urea	5.8 ± 1.2 (17)	5.9 ± 1.2 (12)	5.5 ± 1.3 (5)	0.51
		9.9 ± 3.8 (17)	10.3 ± 4.4 (12)	8.9 ± 1.7 (5)	0.49
	Creatinine	3.6 ± 0.9 (17)	3.4 ± 0.8 (12)	4.1 ± 1.1 (5)	0.15
		6.2 ± 2.4 (17)	6.0 ± 2.6 (12)	6.7 ± 1.7 (5)	0.58
	Phosphate	3.0 ± 0.8 (17)	2.8 ± 0.6 (12)	3.5 ± 1.1 (5)	0.11
		5.1 ± 2.0 (17)	4.9 ± 2.2 (12)	5.6 ± 1.3 (5)	0.49
	Potassium	7.5 ± 1.4 (170	8.0 ± 1.2 (12)	6.1 ± 0.8 (5)	0.004
		12.8 ± 5.0 (17)	13.8 ± 5.3 (12)	10.3 ± 3.3 (5)	0.19
D/P ratio at 4 h	Urea	0.66 ± 0.14 (20)	0.60 ± 0.12 (13)	0.78 ± 0.08 (7)	<0.001
	Creatinine	0.41 (0.36–0.53) (20)	0.38 ± 0.05 (13)	0.58 ± 0.12 (7)	0.005
Others	D/D0 at 4 h	0.65 (0.58–0.70) (20)	0.65 ± 0.05 (13)	0.57 ± 0.15 (7)	0.20
	UFV (mL)	160 ± 322 (17)	275 ± 262 (12)	−117 ± 300 (5)	0.02

Data are presented as mean ± SD (*n*) or median (IQR) (*n*). Unpaired *t*-test with or without Welch’s correction (if appropriate) was used to compare data during peritonitis versus no peritonitis. MTAC = mass transfer area coefficient (calculated with simplified Garred formula), D/P ratio = ratio of dialysate and plasma concentration, D/D0 = ratio of dialysate glucose concentration at end and start of the dwell UFV = ultrafiltration volume after 4 h.

## Data Availability

The data presented in this study are available in this article or supplementary material.

## Supplementary Materials

The following supporting information can be downloaded at: https://www.mdpi.com/article/10.3390/toxins14090635/s1, Figure S1: C4d staining; Figure S2: individual creatinine curves (lifetime); Figure S3: animal body weight; Figure S4: individual urea and creatinine curves (acute-on-chronic kidney injury).
