# Supplementary material for: A Uremic Pig Model for Peritoneal Dialysis
Source: Toxins (Basel). 2022 Sep 14;14(9):635. doi: 10.3390/toxins14090635 (PMC9503030; doi:10.3390/toxins14090635)
Supplement: Supplementary file 1 [file toxins-14-00635-s001.zip › toxins-1871645-supplementary.pdf]

toxins

Supplementary Material: A Uremic Pig Model for Peritoneal Dialysis

Joost C. de Vries, Maaike K. van Gelder, Anneke S. Monninkhof, Sabbir Ahmed, Diënty H. M. Hazenbrink, Tri Q. Nguyen, Gèrard A. P. de Kort, Evert-Jan P. A. Vonken, Koen R. D. Vaessen, Jaap A. Joles, Marianne C. Verhaar and Karin G. F. Gerritsen

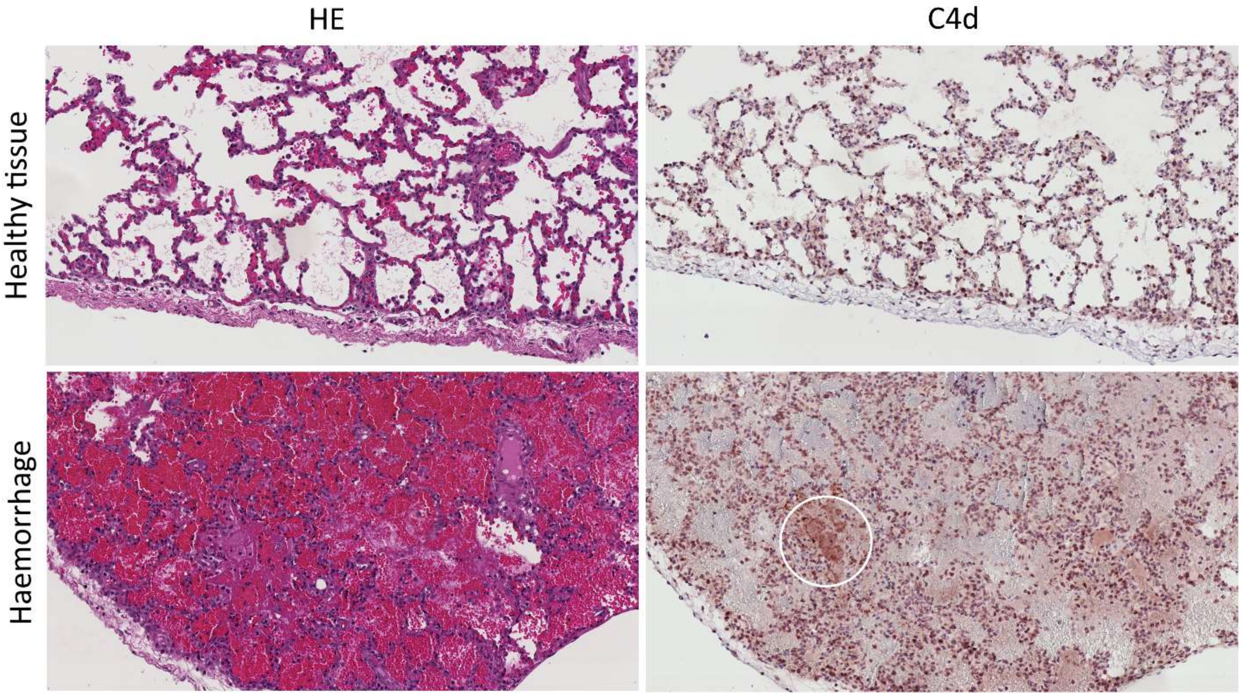

Figure S1. HE (left panels) and C4d (right panels) stain of lung tissue of the two animals that died post embolization. Haemorrhagic lung tissue showed small C4d positive patches (white circle), that were absent in the non-affected areas of the lung.

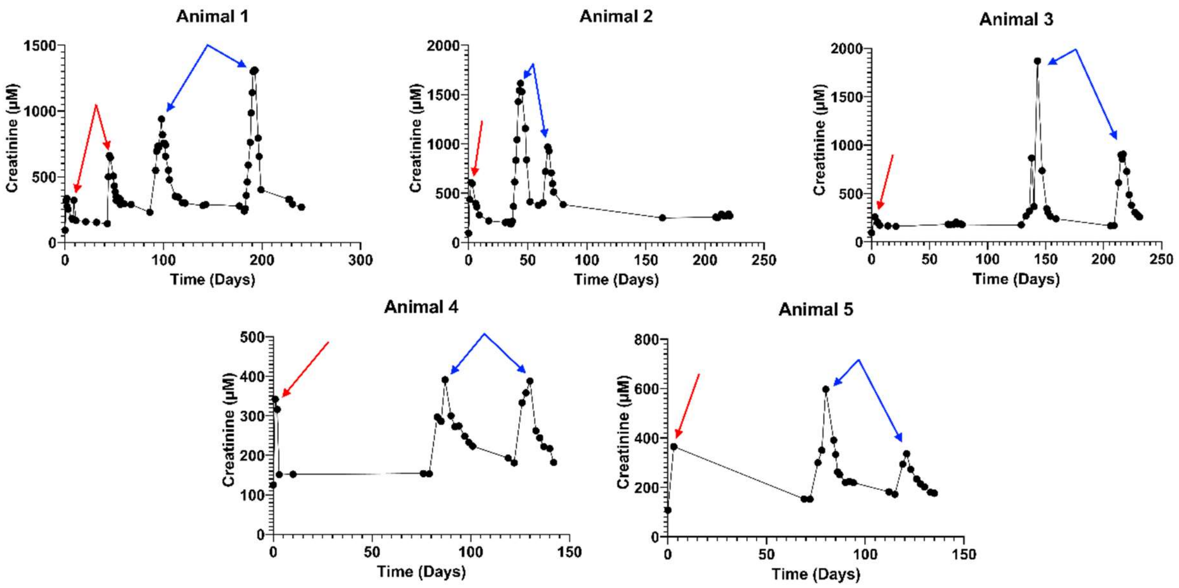

Figure S2 Creatinine concentrations during follow-up of all individual animals. Red arrows indicate the embolization, the blue arrows indicate an episode of gentamicin-induced acute-on-chronic kidney injury.

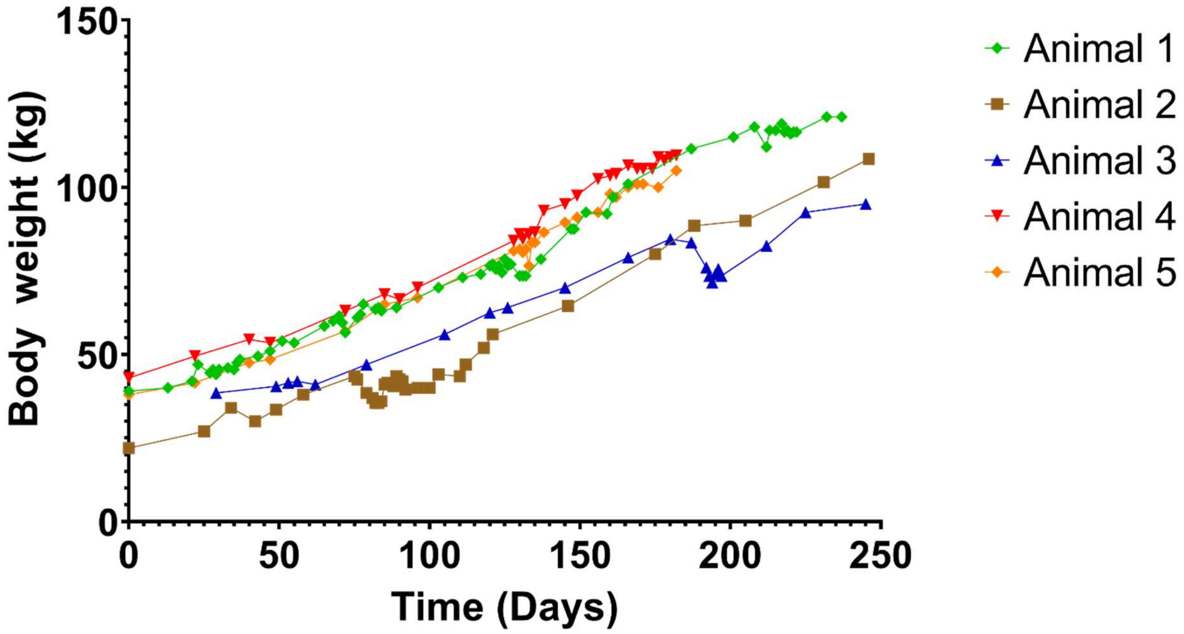

Figure S3. Body weight of the animals during follow-up.

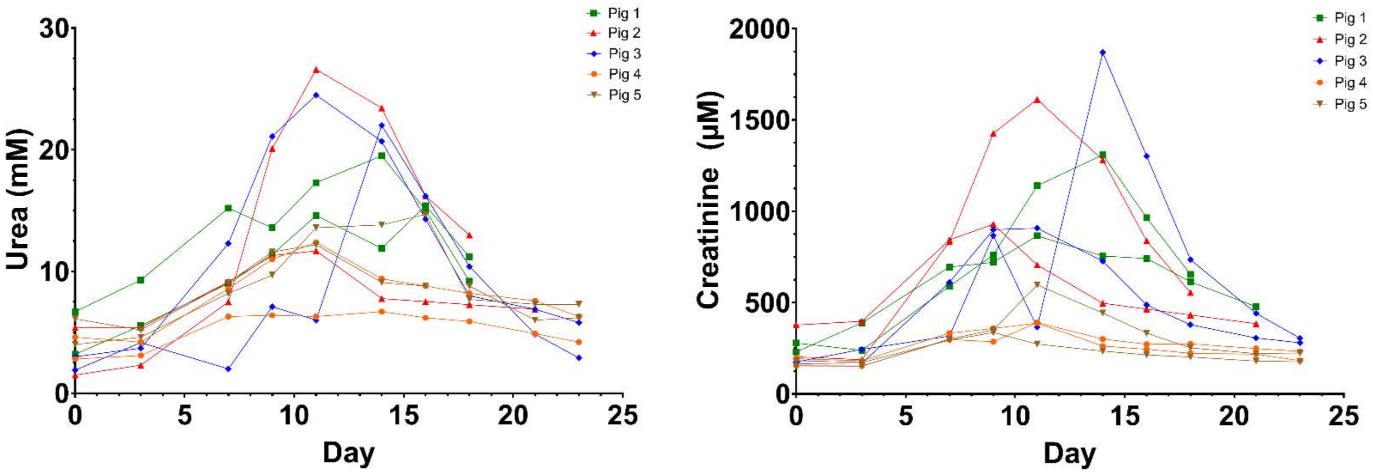

Figure S4. Urea and creatinine curves during acute-on-chronic kidney injury; each line represents an individual animal.
